# Supplementary material for: BRAFV600E Expression in Thyrocytes Causes Recruitment of Immunosuppressive STABILIN-1 Macrophages
Source: Cancers (Basel). 2022 Sep 26;14(19):4687. doi: 10.3390/cancers14194687 (PMC9563029; doi:10.3390/cancers14194687)
Supplement: Supplementary file 1 [file cancers-14-04687-s001.zip › cancers-1861759-supplementary.pptx]

## Slide 1
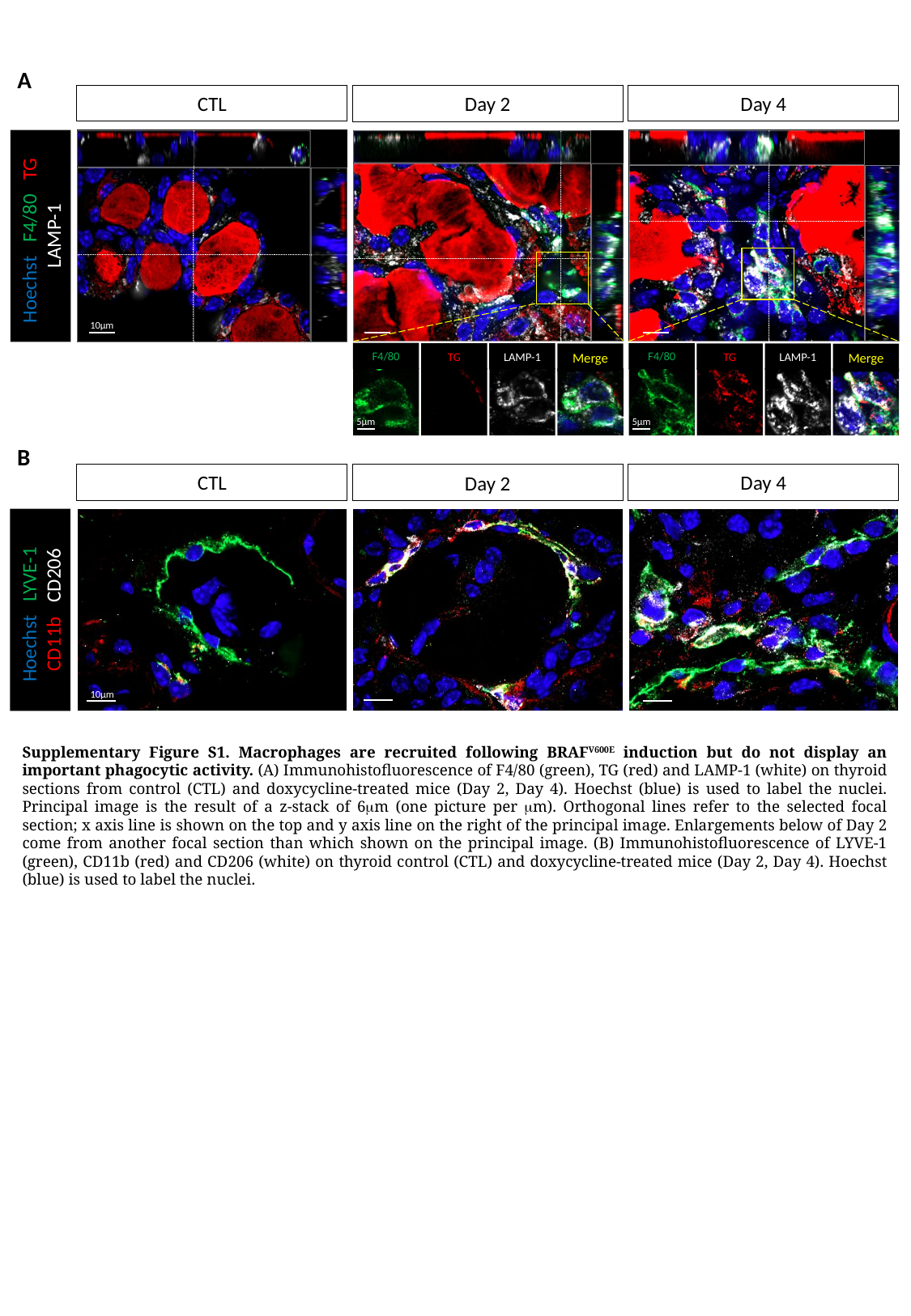

A
Day 4
CTL
Day 2
Hoechst F4/80 TG LAMP-1
10µm
F4/80
F4/80
TG
LAMP-1
Merge
LAMP-1
TG
Merge
5µm
5µm
B
Day 4
CTL
Day 2
Hoechst LYVE-1 CD11b CD206
10µm
Supplementary Figure S1. Macrophages are recruited following BRAFV600E induction but do not display an important phagocytic activity. (A) Immunohistofluorescence of F4/80 (green), TG (red) and LAMP-1 (white) on thyroid sections from control (CTL) and doxycycline-treated mice (Day 2, Day 4). Hoechst (blue) is used to label the nuclei. Principal image is the result of a z-stack of 6μm (one picture per μm). Orthogonal lines refer to the selected focal section; x axis line is shown on the top and y axis line on the right of the principal image. Enlargements below of Day 2 come from another focal section than which shown on the principal image. (B) Immunohistofluorescence of LYVE-1 (green), CD11b (red) and CD206 (white) on thyroid control (CTL) and doxycycline-treated mice (Day 2, Day 4). Hoechst (blue) is used to label the nuclei.

## Slide 2
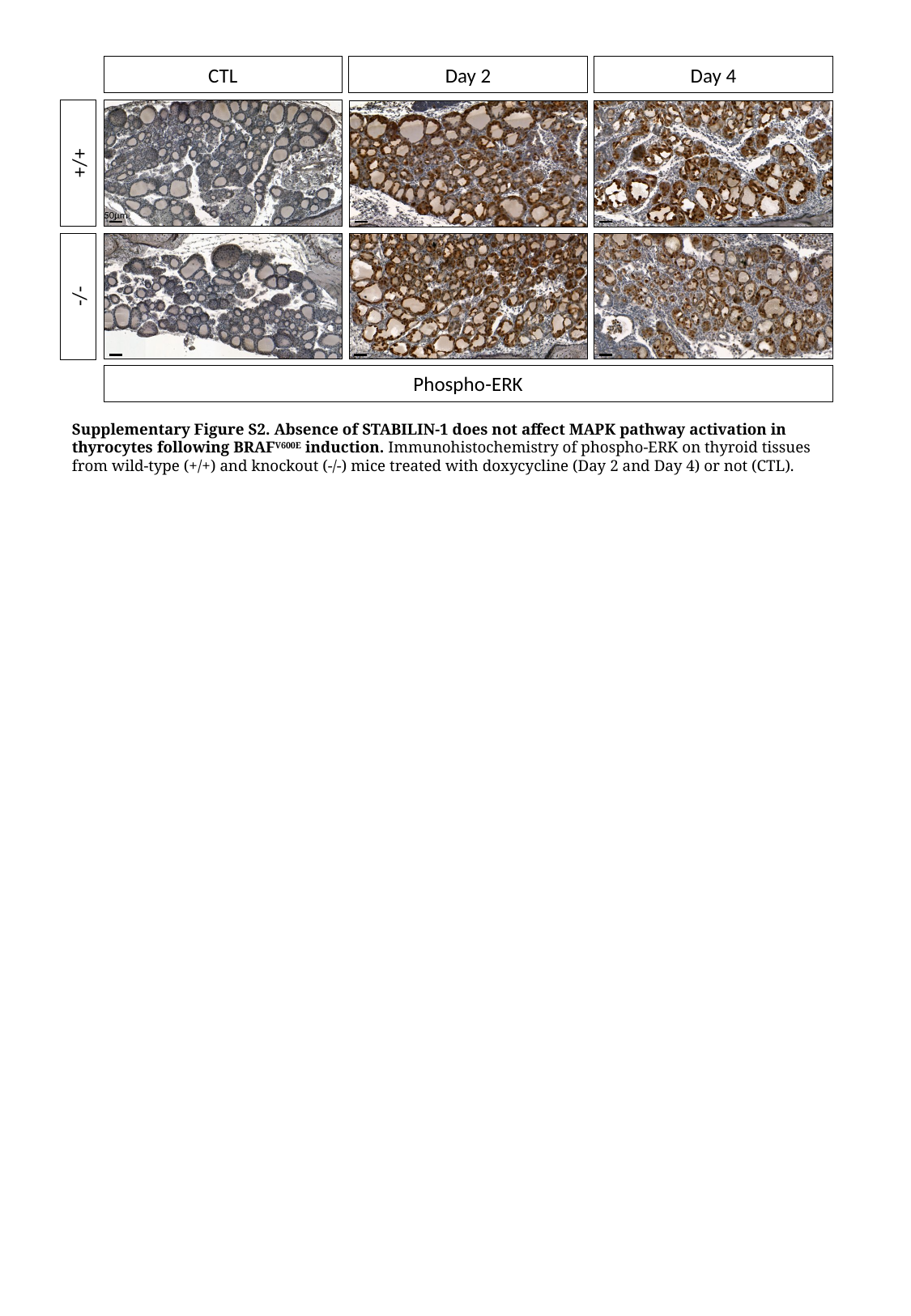

Day 2
Day 4
CTL
+/+
50µm
-/-
Phospho-ERK
Supplementary Figure S2. Absence of STABILIN-1 does not affect MAPK pathway activation in thyrocytes following BRAFV600E induction. Immunohistochemistry of phospho-ERK on thyroid tissues from wild-type (+/+) and knockout (-/-) mice treated with doxycycline (Day 2 and Day 4) or not (CTL).

## Slide 3
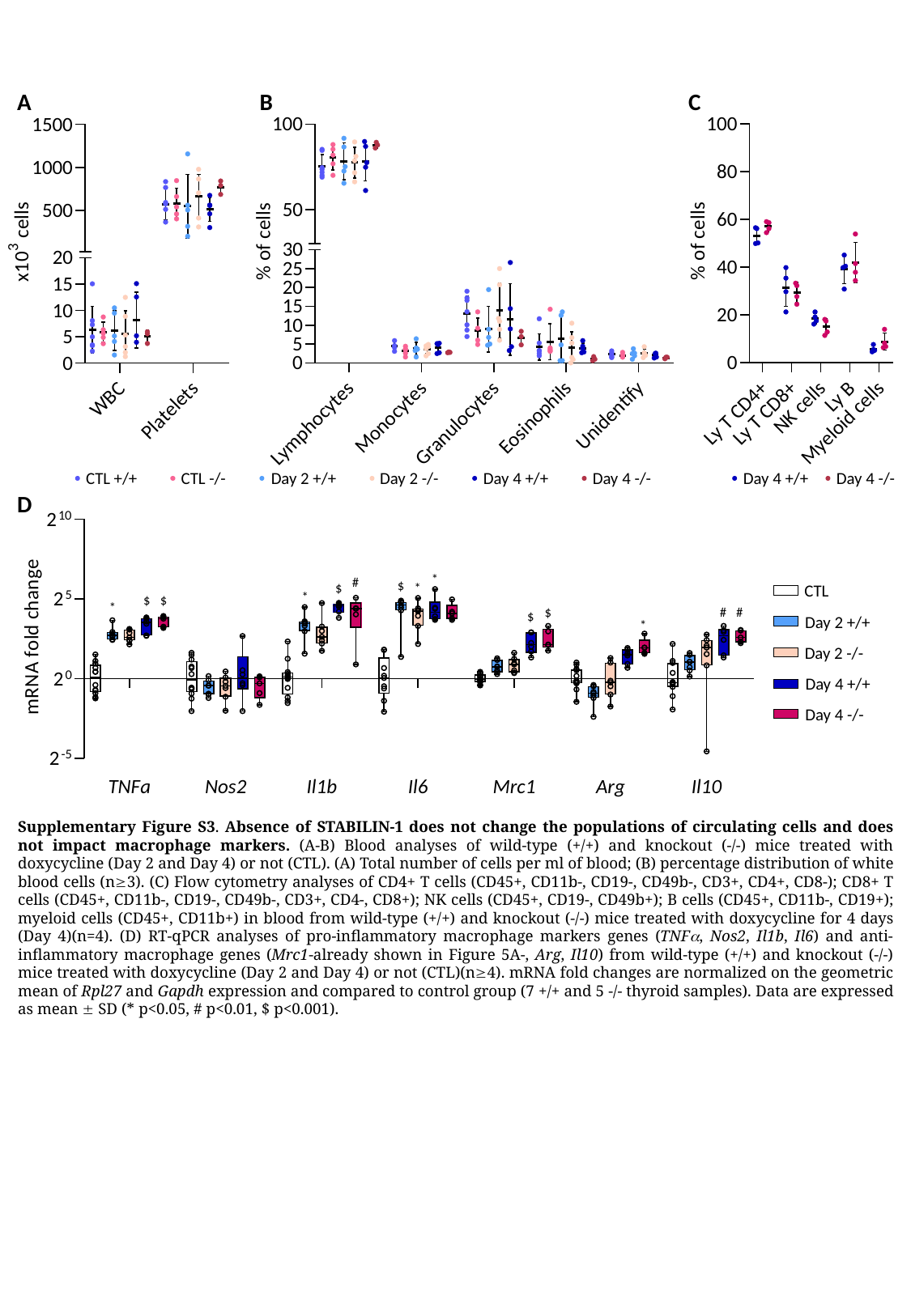

A
B
C
CTL +/+
CTL -/-
Day 2 +/+
Day 2 -/-
Day 4 +/+
Day 4 -/-
Day 4 +/+
Day 4 -/-
D
*
#
$
$
*
CTL
Day 2 +/+
Day 2 -/-
Day 4 +/+
Day 4 -/-
*
$
$
*
$
#
#
$
*
Supplementary Figure S3. Absence of STABILIN-1 does not change the populations of circulating cells and does not impact macrophage markers. (A-B) Blood analyses of wild-type (+/+) and knockout (-/-) mice treated with doxycycline (Day 2 and Day 4) or not (CTL). (A) Total number of cells per ml of blood; (B) percentage distribution of white blood cells (n≥3). (C) Flow cytometry analyses of CD4+ T cells (CD45+, CD11b-, CD19-, CD49b-, CD3+, CD4+, CD8-); CD8+ T cells (CD45+, CD11b-, CD19-, CD49b-, CD3+, CD4-, CD8+); NK cells (CD45+, CD19-, CD49b+); B cells (CD45+, CD11b-, CD19+); myeloid cells (CD45+, CD11b+) in blood from wild-type (+/+) and knockout (-/-) mice treated with doxycycline for 4 days (Day 4)(n=4). (D) RT-qPCR analyses of pro-inflammatory macrophage markers genes (TNFα, Nos2, Il1b, Il6) and anti-inflammatory macrophage genes (Mrc1-already shown in Figure 5A-, Arg, Il10) from wild-type (+/+) and knockout (-/-) mice treated with doxycycline (Day 2 and Day 4) or not (CTL)(n≥4). mRNA fold changes are normalized on the geometric mean of Rpl27 and Gapdh expression and compared to control group (7 +/+ and 5 -/- thyroid samples). Data are expressed as mean ± SD (* p<0.05, # p<0.01, $ p<0.001).

## Slide 4
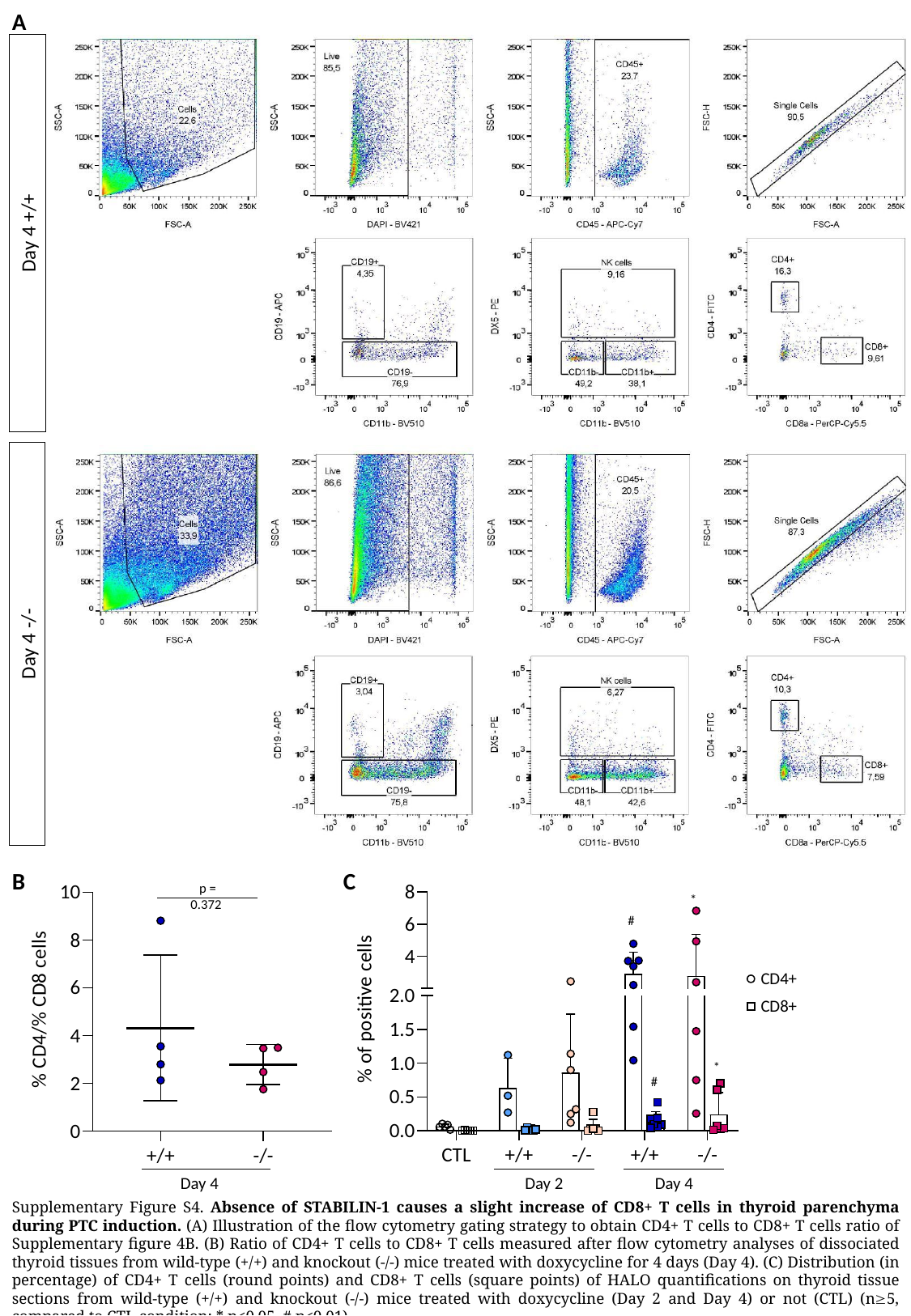

A
Day 4 +/+
Day 4 -/-
B
C
p = 0.372
*
#
CD4+
CD8+
*
#
Day 4
Day 2
Day 4
Supplementary Figure S4. Absence of STABILIN-1 causes a slight increase of CD8+ T cells in thyroid parenchyma during PTC induction. (A) Illustration of the flow cytometry gating strategy to obtain CD4+ T cells to CD8+ T cells ratio of Supplementary figure 4B. (B) Ratio of CD4+ T cells to CD8+ T cells measured after flow cytometry analyses of dissociated thyroid tissues from wild-type (+/+) and knockout (-/-) mice treated with doxycycline for 4 days (Day 4). (C) Distribution (in percentage) of CD4+ T cells (round points) and CD8+ T cells (square points) of HALO quantifications on thyroid tissue sections from wild-type (+/+) and knockout (-/-) mice treated with doxycycline (Day 2 and Day 4) or not (CTL) (n≥5, compared to CTL condition; * p<0.05, # p<0.01).

## Slide 5
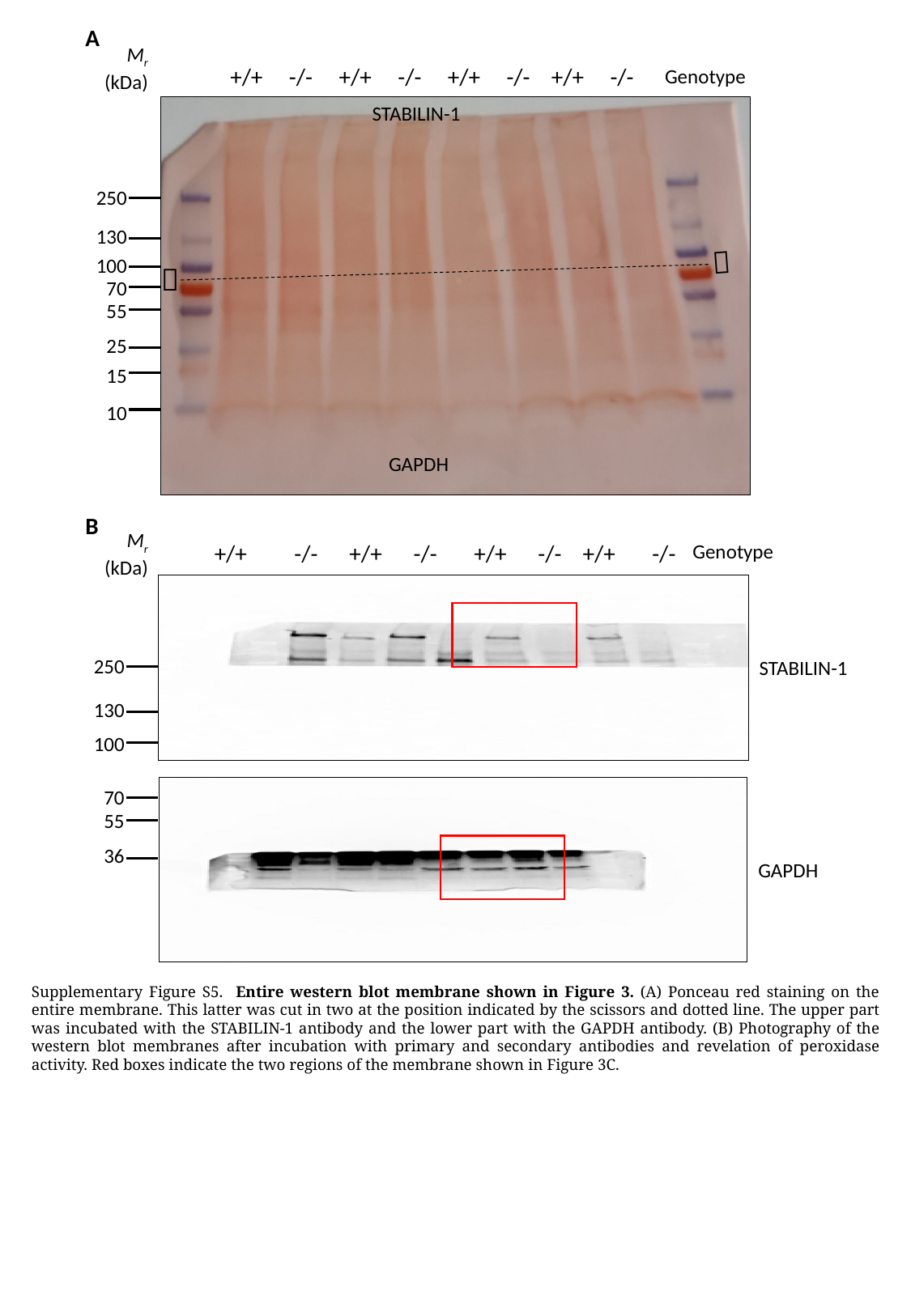

A
Mr
(kDa)
+/+ -/- +/+ -/- +/+ -/- +/+ -/-
Genotype
STABILIN-1
250
130

100

70
55
25
15
10
GAPDH
B
Mr
(kDa)
+/+ -/- +/+ -/- +/+ -/- +/+ -/-
Genotype
250
STABILIN-1
130
100
70
55
36
GAPDH
Supplementary Figure S5. Entire western blot membrane shown in Figure 3. (A) Ponceau red staining on the entire membrane. This latter was cut in two at the position indicated by the scissors and dotted line. The upper part was incubated with the STABILIN-1 antibody and the lower part with the GAPDH antibody. (B) Photography of the western blot membranes after incubation with primary and secondary antibodies and revelation of peroxidase activity. Red boxes indicate the two regions of the membrane shown in Figure 3C.

## Slide 6
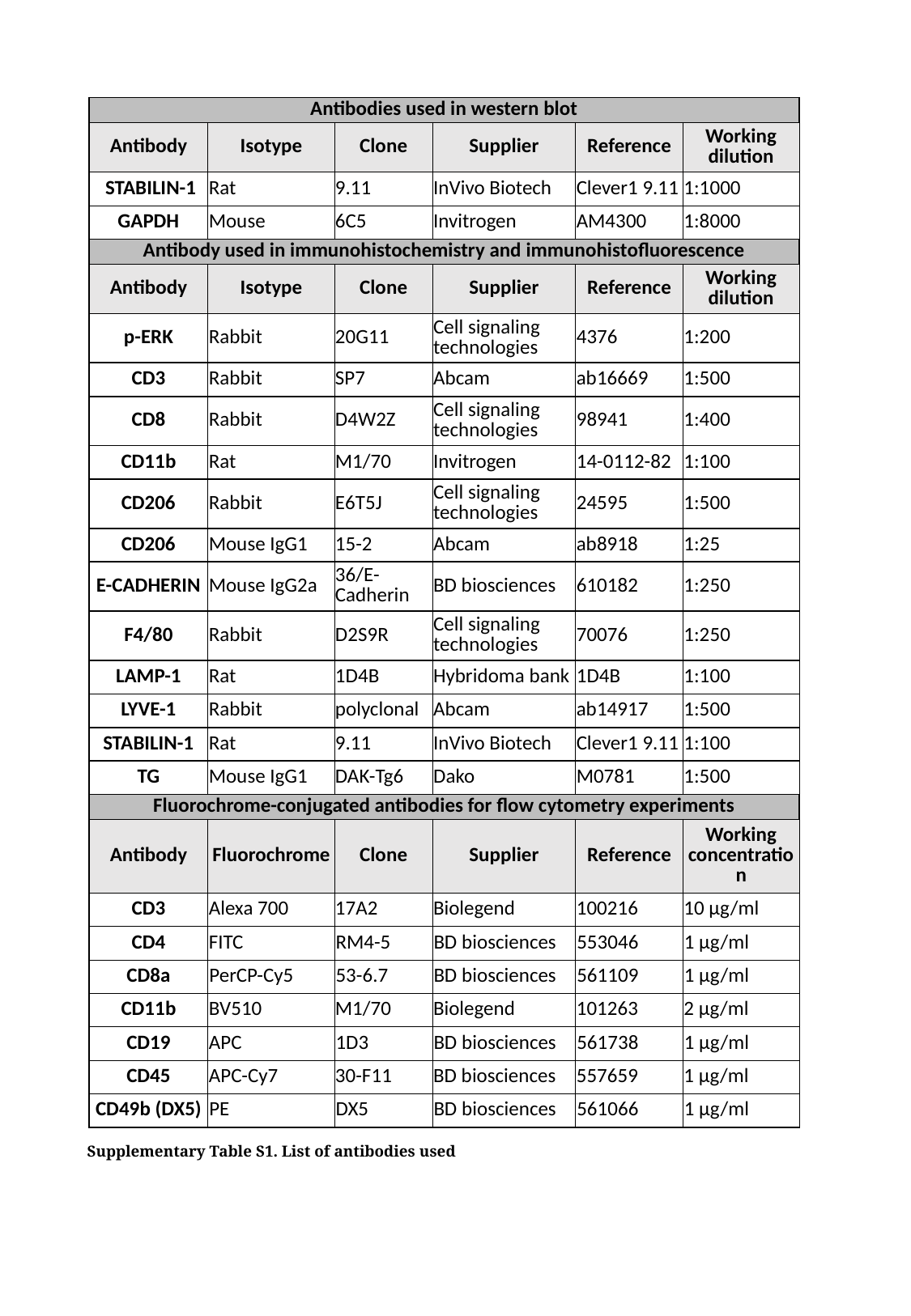

| Antibodies used in western blot | | | | | |
| --- | --- | --- | --- | --- | --- |
| Antibody | Isotype | Clone | Supplier | Reference | Working dilution |
| STABILIN-1 | Rat | 9.11 | InVivo Biotech | Clever1 9.11 | 1:1000 |
| GAPDH | Mouse | 6C5 | Invitrogen | AM4300 | 1:8000 |
| Antibody used in immunohistochemistry and immunohistofluorescence | | | | | |
| Antibody | Isotype | Clone | Supplier | Reference | Working dilution |
| p-ERK | Rabbit | 20G11 | Cell signaling technologies | 4376 | 1:200 |
| CD3 | Rabbit | SP7 | Abcam | ab16669 | 1:500 |
| CD8 | Rabbit | D4W2Z | Cell signaling technologies | 98941 | 1:400 |
| CD11b | Rat | M1/70 | Invitrogen | 14-0112-82 | 1:100 |
| CD206 | Rabbit | E6T5J | Cell signaling technologies | 24595 | 1:500 |
| CD206 | Mouse IgG1 | 15-2 | Abcam | ab8918 | 1:25 |
| E-CADHERIN | Mouse IgG2a | 36/E-Cadherin | BD biosciences | 610182 | 1:250 |
| F4/80 | Rabbit | D2S9R | Cell signaling technologies | 70076 | 1:250 |
| LAMP-1 | Rat | 1D4B | Hybridoma bank | 1D4B | 1:100 |
| LYVE-1 | Rabbit | polyclonal | Abcam | ab14917 | 1:500 |
| STABILIN-1 | Rat | 9.11 | InVivo Biotech | Clever1 9.11 | 1:100 |
| TG | Mouse IgG1 | DAK-Tg6 | Dako | M0781 | 1:500 |
| Fluorochrome-conjugated antibodies for flow cytometry experiments | | | | | |
| Antibody | Fluorochrome | Clone | Supplier | Reference | Working concentration |
| CD3 | Alexa 700 | 17A2 | Biolegend | 100216 | 10 µg/ml |
| CD4 | FITC | RM4-5 | BD biosciences | 553046 | 1 µg/ml |
| CD8a | PerCP-Cy5 | 53-6.7 | BD biosciences | 561109 | 1 µg/ml |
| CD11b | BV510 | M1/70 | Biolegend | 101263 | 2 µg/ml |
| CD19 | APC | 1D3 | BD biosciences | 561738 | 1 µg/ml |
| CD45 | APC-Cy7 | 30-F11 | BD biosciences | 557659 | 1 µg/ml |
| CD49b (DX5) | PE | DX5 | BD biosciences | 561066 | 1 µg/ml |
Supplementary Table S1. List of antibodies used

## Slide 7
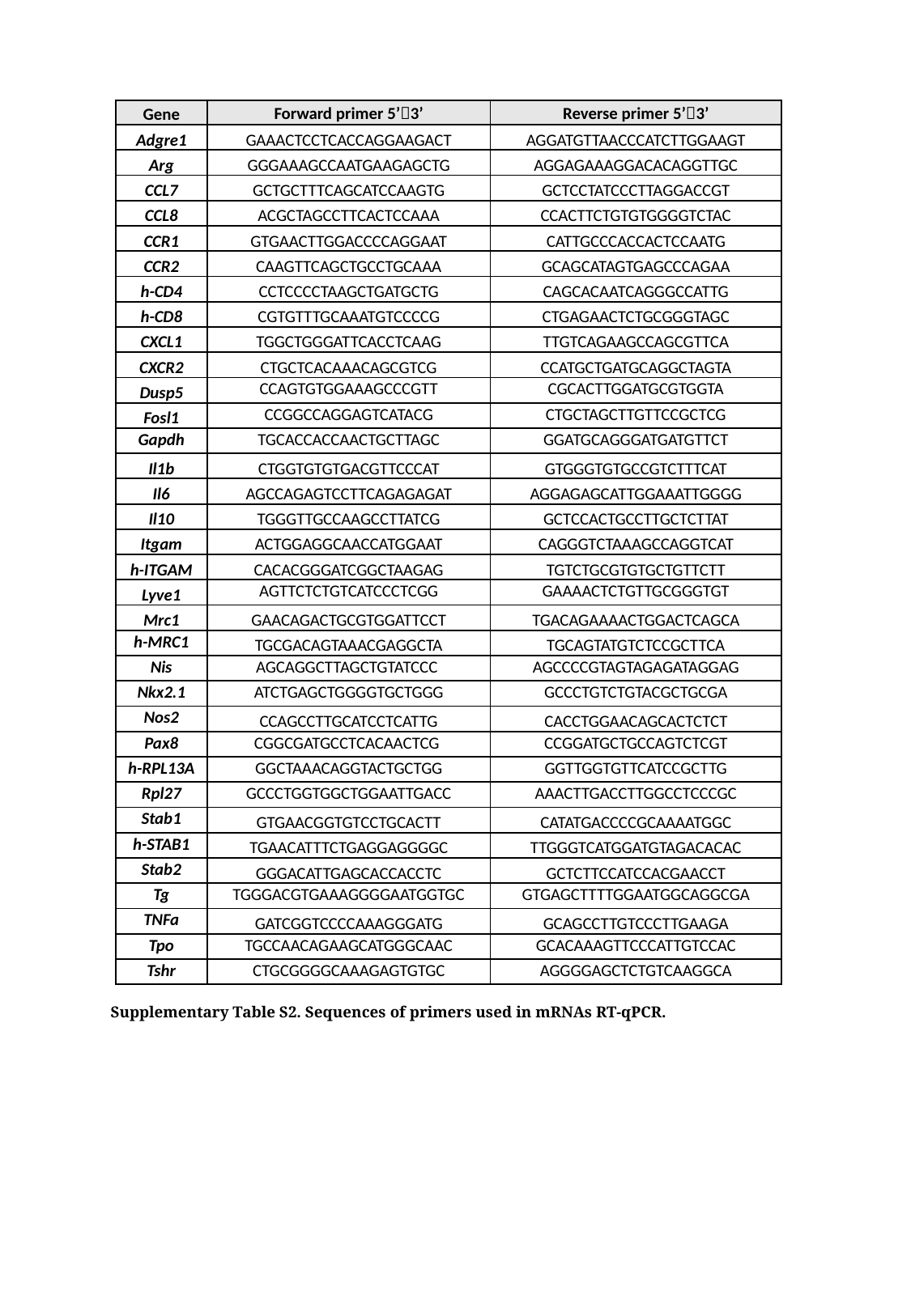

| Gene | Forward primer 5’3’ | Reverse primer 5’3’ |
| --- | --- | --- |
| Adgre1 | GAAACTCCTCACCAGGAAGACT | AGGATGTTAACCCATCTTGGAAGT |
| Arg | GGGAAAGCCAATGAAGAGCTG | AGGAGAAAGGACACAGGTTGC |
| CCL7 | GCTGCTTTCAGCATCCAAGTG | GCTCCTATCCCTTAGGACCGT |
| CCL8 | ACGCTAGCCTTCACTCCAAA | CCACTTCTGTGTGGGGTCTAC |
| CCR1 | GTGAACTTGGACCCCAGGAAT | CATTGCCCACCACTCCAATG |
| CCR2 | CAAGTTCAGCTGCCTGCAAA | GCAGCATAGTGAGCCCAGAA |
| h-CD4 | CCTCCCCTAAGCTGATGCTG | CAGCACAATCAGGGCCATTG |
| h-CD8 | CGTGTTTGCAAATGTCCCCG | CTGAGAACTCTGCGGGTAGC |
| CXCL1 | TGGCTGGGATTCACCTCAAG | TTGTCAGAAGCCAGCGTTCA |
| CXCR2 | CTGCTCACAAACAGCGTCG | CCATGCTGATGCAGGCTAGTA |
| Dusp5 | CCAGTGTGGAAAGCCCGTT | CGCACTTGGATGCGTGGTA |
| Fosl1 | CCGGCCAGGAGTCATACG | CTGCTAGCTTGTTCCGCTCG |
| Gapdh | TGCACCACCAACTGCTTAGC | GGATGCAGGGATGATGTTCT |
| Il1b | CTGGTGTGTGACGTTCCCAT | GTGGGTGTGCCGTCTTTCAT |
| Il6 | AGCCAGAGTCCTTCAGAGAGAT | AGGAGAGCATTGGAAATTGGGG |
| Il10 | TGGGTTGCCAAGCCTTATCG | GCTCCACTGCCTTGCTCTTAT |
| Itgam | ACTGGAGGCAACCATGGAAT | CAGGGTCTAAAGCCAGGTCAT |
| h-ITGAM | CACACGGGATCGGCTAAGAG | TGTCTGCGTGTGCTGTTCTT |
| Lyve1 | AGTTCTCTGTCATCCCTCGG | GAAAACTCTGTTGCGGGTGT |
| Mrc1 | GAACAGACTGCGTGGATTCCT | TGACAGAAAACTGGACTCAGCA |
| h-MRC1 | TGCGACAGTAAACGAGGCTA | TGCAGTATGTCTCCGCTTCA |
| Nis | AGCAGGCTTAGCTGTATCCC | AGCCCCGTAGTAGAGATAGGAG |
| Nkx2.1 | ATCTGAGCTGGGGTGCTGGG | GCCCTGTCTGTACGCTGCGA |
| Nos2 | CCAGCCTTGCATCCTCATTG | CACCTGGAACAGCACTCTCT |
| Pax8 | CGGCGATGCCTCACAACTCG | CCGGATGCTGCCAGTCTCGT |
| h-RPL13A | GGCTAAACAGGTACTGCTGG | GGTTGGTGTTCATCCGCTTG |
| Rpl27 | GCCCTGGTGGCTGGAATTGACC | AAACTTGACCTTGGCCTCCCGC |
| Stab1 | GTGAACGGTGTCCTGCACTT | CATATGACCCCGCAAAATGGC |
| h-STAB1 | TGAACATTTCTGAGGAGGGGC | TTGGGTCATGGATGTAGACACAC |
| Stab2 | GGGACATTGAGCACCACCTC | GCTCTTCCATCCACGAACCT |
| Tg | TGGGACGTGAAAGGGGAATGGTGC | GTGAGCTTTTGGAATGGCAGGCGA |
| TNFa | GATCGGTCCCCAAAGGGATG | GCAGCCTTGTCCCTTGAAGA |
| Tpo | TGCCAACAGAAGCATGGGCAAC | GCACAAAGTTCCCATTGTCCAC |
| Tshr | CTGCGGGGCAAAGAGTGTGC | AGGGGAGCTCTGTCAAGGCA |
Supplementary Table S2. Sequences of primers used in mRNAs RT-qPCR.
